# Laparoscopic or Open Liver Resection Versus Multibipolar Radiofrequency Ablation of HCC Within Milan Criteria on Cirrhosis

**DOI:** 10.1111/liv.70802

**Published:** 2026-07-20

**Authors:** Christian Hobeika, François Cauchy, Agnès Rode, Ephrem Salamé, Olivier Scatton, Lilian Schwarz, Panteleimon Papadopoulos, Arnaud Hocquelet, Anne‐Frédérique Manichon, Christophe Aubé, Frederic Oberti, Angelo Della Corte, Alain Luciani, Suzanne Gay, Mickaël Lesurtel, Chetana Lim, Pierre Nahon, Nathalie Ganne‐Carrié, Lorraine Blaise, Olivier Soubrane, Olivier Sutter, Raffaele Brustia, Louise Barbier, Alexis Laurent, Olivier Seror, Jean‐Charles Nault

**Affiliations:** ^1^ Department of HPB Surgery and Liver Transplantation Beaujon Hospital, AP‐HP, Clichy, Paris‐Cité University Paris France; ^2^ Department of Hepato‐Pancreato‐Biliary and Liver Transplantation Hôpitaux Universitaires de Genève Genève Switzerland; ^3^ Service de radiologie CHU Hospices Civils de Lyon Lyon France; ^4^ Service de chirurgie Digestive et Transplantation Hépatique CHU Tours, Université de Tours Tours France; ^5^ Service de chirurgie hépatobiliaire et digestive, APHP CHU Pitié Salpétrière Paris France; ^6^ Service de chirurgie hépatobiliaire et digestive CHU de Rouen Rouen France; ^7^ Service de radiologie CHU Bordeaux Bordeaux France; ^8^ Service of Diagnostic and Interventional Radiology Lausanne University Hospital and University of Lausanne Lausanne Switzerland; ^9^ Service de radiologie Hôpital Croix Rousse Lyon France; ^10^ Department of Radiology Angers University Hospital, CHU d'Angers Angers France; ^11^ Laboratoire HIFIH, EA 3859, UNIV Angers Université d'Angers Angers France; ^12^ Service d'imagerie Médicale Hôpital Henri Mondor, APHP, UPEC, Créteil, INSERM U955 – Equipe 18 Créteil France; ^13^ Department of Hepato Gastroenterology Angers University Hospital, CHU d'Angers Angers France; ^14^ Service d'Imagerie Médicale AP‐HP, Hôpitaux Universitaires Henri Mondor, Faculté de Santé, Université Paris Est Créteil. INSERM IMRB Créteil France; ^15^ AP‐HP, hôpital Avicenne, service d'hépatologie Bobigny France; ^16^ Unité de Formation et de Recherche Santé Médecine et Biologie Humaine Université Paris 13, Communauté d'Universités et Etablissements Sorbonne Paris Cité Paris France; ^17^ Centre de Recherche des Cordeliers, Sorbonne Université Inserm, Université de Paris, team « Functional Genomics of Solid Tumors » Paris France; ^18^ Department of Digestive Surgery Institut Mutualiste Montsouris Paris France; ^19^ Unité fonctionnelle de radiologie interventionnelle, APHP Hôpital Avicenne Bobigny France; ^20^ Université Paris‐Est Créteil, UPEC Créteil France; ^21^ Team “Pathophysiology and Therapy of Chronic Viral Hepatitis and Related Cancers”, Department of Digestive and Hepato‐Pancreatic‐Biliary Surgery Assistance Publique‐Hôpitaux de Paris, Henri Mondor‐University Hospital Créteil France; ^22^ Department of HPB Surgery and Transplantation Auckland City Hospital Auckland New Zealand

**Keywords:** cirrhosis, hepatocellular carcinoma, minimally invasive surgery, propensity score matching, radiofrequency ablation, surgery, transplantation

## Abstract

**Background & Aims:**

We compared multibipolar radiofrequency ablation (mbpRFA) with open liver resection (OLR) or laparoscopic liver resection (LLR) in patients with HCCs within Milan.

**Methods:**

This multicenter cohort study included patients treated by mbpRFA, OLR or LLR for HCC within the Milan Criteria, developed on advanced fibrosis in nine French centres between 2008 and 2018. Adjustments on HCC size/number, gender, age, ASA, HCV, HBV, metabolic syndrome, chronic alcohol intake, MELD score, serum AFP level, APRI score, cirrhosis, significant portal hypertension and HCC localization were performed using multivariable regressions with centre‐robust variance and propensity score‐based matching (3:1 nearest‐neighbour with calliper < 0.1; effective ratios 2.18:1 and 2.43:1).

**Results:**

1040 patients were included (median age: 64 years; male 82.9%), with 606, 266 and 168 treated by mbpRFA, LLR and OLR. Most HCCs were solitary (84.8%) and on cirrhosis (89.2%). In months, the follow‐up was 50.2 (95% CI: 45.3, 54.3), 43.3 (95% CI: 40.0, 48.6) and 47.4 (95% CI: 40.8, 58.7) for mbpRFA, LLR and OLR patients. After matching, OLR patients had similar overall survival (OS) (HR = 1.05 [CI 95% = 0.65, 1.69], *p* = 0.853), transplant‐free survival (TFS) (HR = 0.91 [CI 95% = 0.56, 1.47]; *p* = 0.690) or recurrence‐free survival (RFS) (HR = 0.99 [CI 95% = 0.69, 1.43]; *p* = 0.968) compared to mbpRFA patients but with more severe adverse events (RR = 2.58 [CI 95% = 1.16, 5.71]; *p* = 0.02) and mortality (RR = 4.51 [CI 95% = 1.16, 17.59]; *p* = 0.03). After matching, LLR patients had better OS (HR = 0.57 [CI 95% = 0.38, 0.88], *p* = 0.01) but similar TFS (HR = 0.77 [CI 95% = 0.55, 1.1]; *p* = 0.152) and more severe adverse events (RR = 2.61 [CI 95% = 1.15, 5.96]; *p* = 0.022) compared to mbpRFA patients. LLR patients had a better RFS (HR = 0.7 [CI 95% = 0.53, 0.91]; *p* = 0.009) than mbpRFA patients. When distant‐only recurrences were considered in the mbpRFA patients, there was no significant difference in RFS (*p* = 0.087). The LLR survival benefit was consistent across pre‐specified subgroups, with no interaction surviving correction for multiple testing.

**Conclusions:**

Minimally invasive modalities (LLR or mbpRFA) should be prioritized when treating early HCC. LLR maximizes oncological outcomes, but mbpRFA maintains optimal TFS with less morbidity.

## Key Points

- Laparoscopic liver resection (LLR) achieved better overall survival and recurrence‐free survival than multibipolar radiofrequency ablation (mbpRFA), with similar transplant‐free survival.
- Open liver resection (OLR) provided oncological outcomes similar to mbpRFA but was associated with more severe adverse events and higher mortality.
- mbpRFA maintained optimal transplant‐free survival with less morbidity, making it a safe first‐line option, particularly in patients with more advanced liver disease.
- Minimally invasive modalities (LLR or mbpRFA) should be prioritized over open surgery when treating early HCC within Milan criteria on cirrhosis.

## Lay Summary

In this large multicenter cohort of patients with early hepatocellular carcinoma developed on advanced fibrosis or cirrhosis, three first‐line curative treatments were compared: multibipolar radiofrequency ablation (mbpRFA), laparoscopic liver resection (LLR), and open liver resection (OLR). Laparoscopic resection achieved better survival than ablation, with similar transplant‐free survival but more severe complications. Open resection had oncological outcomes comparable to ablation but higher severe adverse events and mortality, without survival benefit. These findings support prioritizing minimally invasive approaches over open surgery, with the choice guided by the trade‐off between oncological control and morbidity.

## 1 Introduction

Curative treatments for early hepatocellular carcinoma (HCC) primarily include percutaneous ablation, liver resection and liver transplantation [1], the latter addressing both the cancer and the underlying liver disease [2]. However, its applicability is limited to selected patients due to frequent contraindications, such as comorbidity, age, alcohol consumption and considerations regarding graft shortage and dropout from the waiting list [3]. In contrast, a first‐line treatment by liver resection and radiofrequency ablation can be applied almost immediately, with the option of salvage liver transplantation [4, 5]. Liver resection requires that patients have preserved liver function without significant portal hypertension, especially in the case of major resection [6] while percutaneous radiofrequency ablation is feasible in a broader range of patients [7].

BCLC 0‐A patients are generally considered suitable for both approaches. While it is accepted that ablation and resection achieve similar local tumour control for HCCs ≤ 2 cm, surgery remains the commonly preferred option, especially for tumours above 2 cm or for candidates eligible for transplant [1]. Several randomized controlled trials (RCTs) are currently available, and one only shows the superiority of liver resection compared to ablation [8, 9, 10, 11, 12]. These RCTs have limitations, such as the utilization of monopolar radiofrequency, and included mainly Asian patients. Several observational studies, which also exhibited substantial heterogeneity regarding indications and techniques, have shown that, compared to surgery, ablation can offer similar long‐term results for very early HCC (< 2 cm) in patients with cirrhosis, along with a reduced rate of treatment‐related morbidity [13, 14, 15, 16].

As represented by laparoscopic (LLR) or, more recently, robotic approaches, liver surgeons have embraced minimal‐invasive surgery, as it has been shown to reduce morbidity and hospital stays and improve quality of life [17, 18, 19, 20]. LLR is a new reference standard for easily accessible and unique liver tumours [21, 22]. In this study, we focused on multi‐bipolar radiofrequency ablation (mbpRFA), using intra‐tumour or ‘no‐touch’ techniques, because this method improved the energy deposition, securing optimal ablation area and margins [23], leading to an optimal local control even for large tumours (3–5 cm) [15, 24].

In this observational multicentric study, we aim to compare the short‐ and long‐term outcomes in a European population of patients with HCC within Milan developed on advanced fibrosis/cirrhosis, first‐line treated either by open liver resection (OLR) or laparoscopic liver resection (LLR) to those treated with multi‐bipolar percutaneous radiofrequency ablation (mbpRFA).

## 2 Materials and Methods

### 2.1 Inclusion Criteria

This retrospective multicentric study included patients treated for HCC between January 1, 2008 and December 31, 2018, in one of the following centres in France: Angers, Bobigny, Bordeaux, and Lyon Rouen, Créteil, Pitié‐Salpétrière, Beaujon and Tours (see flow chart Figure S1).

The following inclusion criteria were used:

1. Diagnosis of HCC either by histological analysis or non‐invasive criteria according to the EASL guidelines [25].
2. Within Milan criteria (1 nodule of less than 5 cm or up to 3 nodules of maximum 3 cm);
3. With a first diagnosis of HCC (no previous HCC treatment);
4. Developed on advanced fibrosis defined by F3 at histology on surgical samples or liver biopsy or cirrhosis defined by F4 at histology on surgical samples or liver biopsy or using a combination of non‐invasive biological, clinical and radiological criteria appreciated by the clinician;
5. Treated using mbpRFA or liver resection (either by an open or laparoscopic approach).

### 2.2 Data Collection

Treatment allocation was based on a multidisciplinary tumour board decision at each participating centre, integrating tumour characteristics (size, number, location), liver function (Child‐Pugh, MELD), portal hypertension, comorbidities (ASA), age, and patient preference. The following data were recorded before the treatment of the HCC: demographic variables, ASA score, aetiology of the underlying liver disease, serum AFP level, MELD score, Child Pugh Score, APRI score, fibrosis grade (F3 vs. F4) and the presence of clinically significant portal hypertension (CSPH). The latter was defined as patients having an available Hepatic venous pressure gradient (HVPG) measurement ≥ 10 mmHg or varices recorded by upper endoscopy and/or splenomegaly at imaging and/or low platelet counts < 100 000/mm^3^, according to the data available in each centre. Tumour size, number, HCC localization, and serum AFP levels were collected through biology tests and imaging less than 1 month from the first‐line treatment.

Investigators of this study hypothesize that HCC localization is likely affecting first‐line modality treatment assignment. Therefore, a three‐level HCC localization variable was considered as follows: posterosuperior segments (if one or more HCCs involved segments I, IVa, VII or VIII), anterior segments (including segments IVb, V or VI), and the left lobe (if involving segments II or III). Based on knowledge derived from surgical difficulty scoring systems [26, 27], posterosuperior localizations were considered the most surgically challenging, and left lobe localization likely the most accessible. In the case of multiple HCCs, the most challenging (posterosuperior > anterior > left lobe) localization was considered the main HCC localization [28].

Pathological analysis was collected postoperatively for patients undergoing liver resection (OLR or LLR). It included WHO grade, microvascular invasion, satellite nodules, and the resection margin. Post‐treatment complications were recorded at 90 days of follow‐up and classified from 0 to V (i.e., death) using the Dindo‐Clavien classification [29]. Severe complications were defined as complications graded Dindo‐Clavien ≥ III. Treatment‐related death was defined as death from all causes in the 90 days following the treatment.

### 2.3 Multi‐Bipolar Percutaneous Radiofrequency Ablation Procedure

All mbpRFA procedures were performed under general anaesthesia. MbpRFA was performed percutaneously with imaging guidance [23]. The number of electrodes, energy delivered per treatment, and the application time were not standardized and were based on localization and tumour size. The following data were collected: date of treatment, number of sessions to achieve complete ablation, occurrence of adverse events, and length of hospitalization.

Complete ablation was defined by no enhancement after mbpRFA at the ablation zone visible during the arterial phase of a CT scan or MRI one month after the treatment. If the ablation was deemed incomplete, mbpRFA was repeated up to three times, provided the patient remained eligible for treatment. If additional procedures could not be performed or did not achieve complete ablation, the case was considered a failure of mbpRFA, and the patient was considered as having a local tumour recurrence.

### 2.4 Liver Resection

The surgical approach for liver resection was recorded as OLR or LLR. Major liver resections were defined as hepatectomies involving ≥ 3 consecutive Couinaud's segments [30]. The difficulty of the hepatectomy was graded using IMM classification, which has been validated for both open and laparoscopic approaches [31, 32]. This classification has three levels of difficulty: grade 1 (low difficulty), including small wedge resections (< 3 cm) and left lateral sectionectomy; grade 2 (intermediate difficulty), including anterolateral segmentectomy and left hepatectomy; and grade 3 (high difficulty), including posterosuperior segmentectomy, right posterior sectionectomy, right hepatectomy, extended right hepatectomy, central hepatectomy and extended left hepatectomy.

The surgical indication, approach or technique was not standardized across centres. All enrolled centres adhered to the principle of anatomic resection to remove entirely the tumour‐bearing portal branches bordered by landmark veins whenever possible [33]. Margins were considered negative when ≥ 1 cm. All surgical centres were French expert tertiary centres in HPB and laparoscopic surgery, as they participated in the AFC‐LLR‐2018 study group [34].

### 2.5 Follow‐Up and Right‐Censored Outcomes

The end of the follow‐up was December 2021. Ninety‐day post‐treatment adverse events were defined according to Dindo‐Clavien classification, with those graded ≥ III considered severe [29].

The starting point for all time‐dependent outcomes was the date of the first mbpRFA session in case of repeated ablations, anchoring follow‐up to the first treatment exposure rather than to the date of confirmed complete ablation. This design avoids immortal‐time bias.

Overall survival (OS) was measured as the time from the start of treatment until death (any cause). Recurrence‐free survival (RFS) was defined as the time from the start of treatment to either disease recurrence or death. Time to liver transplantation (TLT) was calculated as the duration until liver transplantation. Transplantation‐free survival (TFS) is the time from the start of treatment to either liver transplantation or death, recurrence being considered as a non‐event.

Time to recurrence (TTR) was defined as the duration until disease recurrence, with patients who underwent liver transplantation (LT) or died before recurrence being censored: liver transplantation and death were treated as competing risks. The reported hazard ratios were cause‐specific hazard ratios, and cumulative incidence functions accounting for competing events were used for the graphical display of recurrence.

The investigators of this study also decided to explore a scenario with an alternative endpoint for recurrence, excluding local recurrences and considering distant‐only ones as an event in the mbpRFA arm [5].

### 2.6 Statistical Analysis

Continuous data were expressed as median (25–75 inter‐quartiles), were not categorized, and were compared using the Mann–Whitney *U* test or Kruskal–Wallis test, as appropriate. Categorical data are expressed as percentages and were compared using Pearson's chi‐square test or Fisher's exact test, as appropriate. Statistical significance testing was 2‐sided. Survival probabilities were computed using the Kaplan–Meier estimate and compared using the log‐rank test. A *p*‐value < 0.05 was considered statistically significant for all tests. Missing values are reported in Table 1, and no variables had > 12% of missing values. Right‐censored and binary outcomes regressions were Cox proportional‐hazards regression models and logistic regressions using cluster‐centre robust variance to account for inter‐centre variability.

**TABLE 1:** Descriptive analysis of the study population and according to the type of treatment received.

		Available data	Whole population	mbpRFA population	LLR population	OLR population	*p*
			*N* = 1040	*N* = 606	*N* = 266	*N* = 168	
Gender (male)		1040	862 (82.9%)	502 (82.8%)	224 (84.2%)	136 (81.0%)	0.680
Age (years)		1040	64.32 [57.67, 71.29]	65.03 [57.78, 73.14]	63.02 [57.13, 68.27]	65.01 [57.97, 70.44]	0.005
ASA grade 3 (vs. 2)		1040	327 (31.4%)	208 (34.3%)	74 (27.8%)	45 (26.8%)	0.060
Chronic HCV		1037	346 (33.4%)	187 (31.0%)	105 (39.5%)	54 (32.1%)	0.048
Chronic HBV		1037	125 (12.1%)	48 (8.0%)	47 (17.7%)	30 (17.9%)	< 0.001
Metabolic syndrome		1037	284 (27.4%)	196 (32.5%)	60 (22.6%)	28 (16.7%)	< 0.001
Chronic alcohol intake		1037	529 (51.0%)	344 (57.0%)	120 (45.1%)	65 (38.7%)	< 0.001
MELD score		976	8.00 [7.00, 10.00]	8.50 [7.00, 10.00]	8.00 [7.00, 9.00]	7.00 [6.00, 9.00]	< 0.001
APRI score		948	0.81 [0.50, 1.44]	0.83 [0.53, 1.51]	0.74 [0.46, 1.34]	0.71 [0.46, 1.21]	0.053
HCC size and number	Single HCC of ≤ 2 cm	1037	292 (28.2%)	175 (28.9%)	77 (28.9%)	40 (24.2%)	< 0.001
	Single HCC 2 to 3 cm		317 (30.6%)	193 (31.8%)	79 (29.7%)	45 (27.3%)	
	Single HCC > 3 cm		270 (26.0%)	121 (20.0%)	88 (33.1%)	61 (37.0%)	
	2 or 3 HCCs ≤ 3 cm		158 (15.2%)	117 (19.3%)	22 (8.3%)	19 (11.5%)	
Serum AFP level		918	7.00 [4.00, 24.00]	7.00 [4.00, 20.00]	8.30 [4.00, 34.29]	7.00 [4.00, 50.00]	0.399
F4 (vs. F3) Fibrosis non‐tumour liver		1040	928 (89.2%)	588 (97.0%)	215 (80.8%)	125 (74.4%)	< 0.001
Tumour localization	Postero superior	1040	546 (52.5%)	318 (52.5%)	115 (43.2%)	113 (67.3%)	< 0.001
	Anterior		262 (25.2%)	163 (26.9%)	66 (24.8%)	33 (19.6%)	
	Left lobe		232 (22.3%)	125 (20.6%)	85 (32.0%)	22 (13.1%)	
Major hepatectomy		434	—	—	27 (10.2)	37 (22.0)	0.001
IMM classification (resection only)	Grade 1	434	—	—	94 (35.3)	25 (15.0)	< 0.001
	Grade 2	434	—	—	112 (42.1)	50 (29.9)	
	Grade 3	434	—	—	60 (22.6)	92 (55.1)	
Anatomical resection		434	—	—	117 (44.0)	101 (60.1)	0.001
Microvascular invasion		310	—	—	46 (27.4)	61 (43.0)	0.006
Macrovascular invasion		323	—	—	11 (6.2)	10 (6.9)	0.824
Satellite nodules		316	—	—	39 (22.3)	22 (15.6)	0.153
Poor tumour differentiation (WHO grade 3)		398	—	—	22 (9.0)	12 (7.8)	0.862
Surgical margin (mm)		421	—	—	6.0 [2.0, 10.8]	6.0 [2.0, 15.0]	0.628
R0 resection		421	—	—	223 (86.4)	139 (85.3)	0.774
Clinically significant portal hypertension		1040	334 (32.1%)	258 (42.6%)	58 (21.8%)	18 (10.7%)	< 0.001
Overall adverse events		1032	360 (34.9%)	178 (29.4%)	93 (35.2%)	89 (54.9%)	< 0.001
Dindo‐Clavien classification (90‐day of follow‐up)	No adverse events	1032	672 (65.1%)	428 (70.6%)	171 (64.8%)	73 (45.1%)	< 0.001
	Grade I	1032	158 (15.3%)	99 (16.3%)	40 (15.2%)	19 (11.7%)	
	Grade II	1032	117 (11.3%)	47 (7.8%)	32 (12.1%)	38 (23.5%)	
	Grade III	1032	37 (3.6%)	17 (2.8%)	7 (2.7%)	13 (8.0%)	
	Grade IV	1032	30 (2.9%)	8 (1.3%)	10 (3.8%)	12 (7.4%)	
	Grade V	1032	18 (1.7%)	7 (1.2%)	4 (1.5%)	7 (4.3%)	
Severe adverse events (Dindo‐Clavien > II)		1032	85 (8.2%)	32 (5.3%)	21 (8.0%)	32 (19.8%)	< 0.001
90‐day mortality (Dindo‐Clavien of V)		1032	18 (1.8%)	7 (1.2%)	4 (1.5%)	7 (4.2%)	0.034
Lengh of stay (days)		1018	5.00 [3.00, 7.00]	3.00 [3.00, 5.00]	6.00 [5.00, 9.00]	8.00 [6.00, 12.00]	< 0.001

*Note:* Continuous variables were expressed using median (interquartile range) and dichotomic variable using number (percentages). The statistical analysis was performed between the three columns (MbpRA, LLR and OLR) using non‐parametric Kruskall Wallis test for continuous value and Fisher test or chi‐squared test for categorical values.

Abbreviations: AFP, alpha foetoprotein; APRI, AST to Platelet Ratio Index; ASA, American Society of Anesthesiologists physical status classification; HBV, hepatitis B virus; HCC, hepatocellular carcinoma; HCV, hepatitis C virus; IMM classificiation, Institut Mutualiste Montsouris classification; MELD, model for end stage liver disease.

The variables included in the propensity score were HCC size/number, gender, age, ASA, HCV, HBV, metabolic syndrome, chronic alcohol intake, MELD score, serum AFP level, APRI score, cirrhosis, CSPH and HCC localization. All propensity score‐based matchings were performed using a 3:1 nearest neighbour matching without replacement using distance (probit regression) and a calliper < 0.1 to estimate marginal effects. The balance of the matching variables before and after matching was estimated using standardized mean differences (Figures S2 and S3).

Marginal effects were computed using the g‐computation method. For continuous or binary endpoints, marginal differences or log risk ratios with 95% CI were computed using a weighted linear or logistic regression incorporating matching weights and clustered variance on matching pair membership to account for cluster‐robust standard errors. These regressions included the outcome of interest as the response variable and, as independent variables, the treatment arm, the quantile distribution of the distance and their interaction. Relative risks were obtained for binary variables by exponentiating the marginal log risk ratios. Marginal HRs were estimated using a weighted (incorporating the matching weights) Cox model without covariates (i.e., non‐collapsible HR) with clustered variance on matching pair membership (cluster‐robust standard errors).

C.H. performed all statistical analyses using R statistical software version 4.2.0. The supplementary methods describe detailed methods and statistical analysis.

## 3 Results

### 3.1 Study Population

Nine centres included 1040 patients with a first diagnosis of HCC within Milan criteria during the study period (flow chart of the study in Figure S1). 606 (58%) were treated with percutaneous mbpRFA, 266 (26%) with LLR, and 168 (16%) with OLR. Table 1 details the descriptive analysis of the whole population and according to the treatment arm. In the mbpRFA subgroup, complete ablation was obtained in 604 patients (99%) after one (*n* = 546), two (*n* = 52), or three sessions (*n* = 6). Negative margins were achieved in 223 (86.4%) LLR patients and 139 (85.3%) OLR patients.

Multivariate analysis in Table S1 showed that patients treated by mbpRFA were older, with less frequent chronic hepatitis B, lower serum AFP, smaller HCC, and less frequently localized in the left lobe, but had more cirrhosis and CSPH. Patients treated by LLR were younger, had a lower rate of cirrhosis and CSPH, and their HCCs were more frequently unique and more frequently localized in the left lobe. Patients treated by OLR had a lower MELD score and larger HCC, the latter being more frequently localized in posterosuperior segments and less frequently developed on cirrhosis with less CSPH.

### 3.2 Short‐ and Long‐Term Outcomes in the Unmatched Population

The rates of adverse events, severe adverse events, and mortality were 34.9%, 8.2% and 1.8%. OLR patients were associated with the highest severe adverse events rates (19.8%; *p* < 0.001), mortality (4.2%; *p* = 0.034), and median hospital length of stay (8 days; *p* < 0.001). Table 1 displays post‐treatment outcomes according to treatment arms.

The median follow‐up was 50.2 (95% CI: 45.3, 54.3) months for mbpRFA, 43.3 months (95% CI: 40.0, 48.6) for LLR, and 47.4 months (95% CI: 40.8, 58.7) for OLR.

**FIGURE 1:**
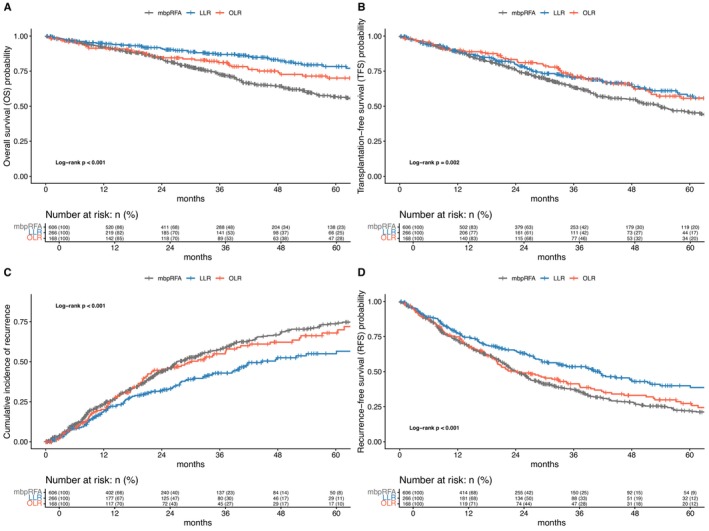
Crude Kaplan–Meier curves in patients treated by mbpRFA versus LLR versus OLR in the study (unmatched) population. Kaplan–Meier curves of (A) Overall survival, (B) Transplant‐free survival, (C) Recurrence‐free survival and (D) Cumulative incidence of recurrence, and in the unmatched population according to the type of treatments received (mbpRFA vs. LLR vs. OLR). Curves were compared using the log‐rank test. The numbers at risk were represented under the *x*‐axis. LLR, laparoscopic liver resection; mbpRFA, multibipolar radiofrequency ablation; OLR, open liver resection.

The 1‐year, 3‐year, and 5‐year rates for OS, RFS, TFS, and TTR in the whole population were as follows: OS‐93%, 78% and 64%; RFS‐74%, 42% and 27%; TFS‐88.9%, 66.2% and 49.9%; and TTR‐78.3%, 46.6% and 31.8%. Local recurrence in mbpRFA occurred in 14.2% (*n* = 86) of the cases, and these tumour local recurrences were retreated efficiently by local ablation in most cases (*n* = 78). Crude comparative survival analyses are displayed in Figure 1. The 5‐year cumulative incidence of transplantation was 15.8% for mbpRFA, 19.5% for OLR and 27.5% for LLR (log‐rank: *p* = 0.020).

Multivariate analyses in Table S2 show factors associated with OS, RFS, TFS, and TLT in the study population. Notably, LLR was independently associated with better RFS (HR: 0.79 [0.65, 0.96]; *p* = 0.017) compared to mbpRFA. Patients with HCC localized in the anterior segments had independently better OS (HR = 0.84 [0.72, 0.97]; *p* = 0.021) and TFS (HR = 0.85 [0.73, 0.98]; *p* = 0.027).

### 3.3 Propensity‐Score Matching Analysis of Patients Treated by LLR vs. mbpRFA

Using propensity‐score‐based analysis, 310 mbpRFA patients were matched to 142 LLR patients. The matched population is described in Table 2. In the matched population, LLR was associated with an increased risk of adverse events (RR = 1.37 [1.02; 1.85]; *p* = 0.039) or severe adverse events (RR = 2.61 [1.15, 5.96]; *p* = 0.022) and with a longer hospital stay (+5 days [3–7]; *p* < 0.001) compared to mbpRFA. LLR was not significantly associated with an increased mortality risk (RR = 3.61 [0.59, 22.24]; *p* = 0.166) compared to mbpRFA.

**TABLE 2:** Descriptive analysis of the matched populations and according to the type of treatment received.

Number of patients		LLR vs. mbpRFA				OLR vs. mbpRFA			
		452	310	142	SMD	240	170	70	SMD
Matching variables^a^		Overall	mbpRFA	LLR		Overall	mbpRFA	OLR	
Single HCC of ≤ 2 cm		129 (28.5)	86 (27.7)	43 (30.3)	0.0200	53 (22.1)	37 (21.8)	16 (22.9)	0.0095
2 cm < Single HCC ≤ 3 cm		131 (29.0)	93 (30.0)	38 (26.8)	−0.0176	75 (31.2)	53 (31.2)	22 (31.4)	0.0071
Single HCC > 3 cm		137 (30.3)	90 (29.0)	47 (33.1)	0.0106	73 (30.4)	50 (29.4)	23 (32.9)	−0.0024
2 or 3 HCCs, ≤ 3 cm		55 (12.2)	41 (13.2)	14 (9.9)	−0.0129	39 (16.2)	30 (17.6)	9 (12.9)	−0.0143
Gender (male)		372 (82.3)	254 (81.9)	118 (83.1)	−0.0035	203 (84.6)	145 (85.3)	58 (82.9)	−0.0310
Age (years)		63.21 [57.03, 69.29]	63.49 [56.86, 71.10]	62.62 [57.86, 68.04]	0.0341	63.27 [57.12, 69.95]	62.80 [56.94, 70.30]	64.41 [58.70, 69.24]	−0.0258
ASA grade 3 (vs. 2)		145 (32.1)	102 (32.9)	43 (30.3)	−0.0305	68 (28.3)	49 (28.8)	19 (27.1)	−0.0262
Chronic HCV		165 (36.5)	111 (35.8)	54 (38.0)	0.0176	81 (33.8)	55 (32.4)	26 (37.1)	0.0405
Chronic HBV		59 (13.1)	37 (11.9)	22 (15.5)	−0.0035	36 (15.0)	24 (14.1)	12 (17.1)	0.0143
Metabolic syndrome		113 (25.0)	81 (26.1)	32 (22.5)	−0.0176	70 (29.2)	51 (30.0)	19 (27.1)	−0.0119
Chronic alcohol intake		244 (54.0)	170 (54.8)	74 (52.1)	−0.0012	114 (47.5)	84 (49.4)	30 (42.9)	−0.0357
MELD score		8.00 [7.00, 10.00]	8.00 [7.00, 10.00]	8.00 [7.00, 9.75]	−0.0216	8.00 [6.00, 9.00]	8.00 [7.00, 9.00]	8.00 [6.00, 9.00]	0.0344
Serum AFP level		7.78 [4.00, 30.25]	7.78 [4.00, 29.75]	7.85 [4.30, 31.42]	−0.0318	7.35 [4.00, 44.25]	8.00 [4.00, 43.00]	7.00 [4.05, 46.25]	−0.0999
APRI score		0.80 [0.50, 1.45]	0.78 [0.50, 1.43]	0.89 [0.53, 1.57]	0.0453	0.74 [0.47, 1.16]	0.72 [0.46, 1.15]	0.80 [0.53, 1.18]	0.0896
F4 fibrosis (vs. F3)		428 (94.7)	296 (95.5)	132 (93.0)	0.0012	219 (91.2)	157 (92.4)	62 (88.6)	0.0333
CSPH		138 (30.5)	101 (32.6)	37 (26.1)	−0.0305	35 (14.6)	27 (15.9)	8 (11.4)	−0.0143
HCC localization	Postero superior	197 (43.6)	137 (44.2)	60 (42.3)	0.0106	155 (64.6)	106 (62.4)	49 (70.0)	0.0714
	Anterior	113 (25.0)	78 (25.2)	35 (24.6)	0.0153	52 (21.7)	38 (22.4)	14 (20.0)	−0.0262
	Left lobe	142 (31.4)	95 (30.6)	47 (33.1)	−0.0258	33 (13.8)	26 (15.3)	7 (10.0)	−0.0452

*Note:* Continuous variables were expressed using median (interquartile range) and dichotomic variable using number (percentages).

Abbreviations: AFP, alpha foetoprotein; APRI, AST to Platelet Ratio Index; ASA, American Society of Anesthesiologists physical status classification; CSPH, Clinically significant portal hypertension; HBV, hepatitis B virus; HCC, hepatocellular; HCV, hepatitis C virus; MELD, model for end stage liver disease; SMD, standard mean differences.

^a^ Matching was performed using a 3:1 (to increase precision) nearest neighbour matching without replacement using propensity score distance (estimated with probit regression) and a calliper < 0.1 to estimate marginal effects (i.e., average effect of exposure on the population). The target estimation was the average treatment effect on the treated. The balance of the matching variables before and after matching was estimated using standardized mean differences. Distances SMDs were 0.0144 and 0.0072 in the LLR vs mbpRFA and OLR vs mbpRFA matches, respectively.

**FIGURE 2:**
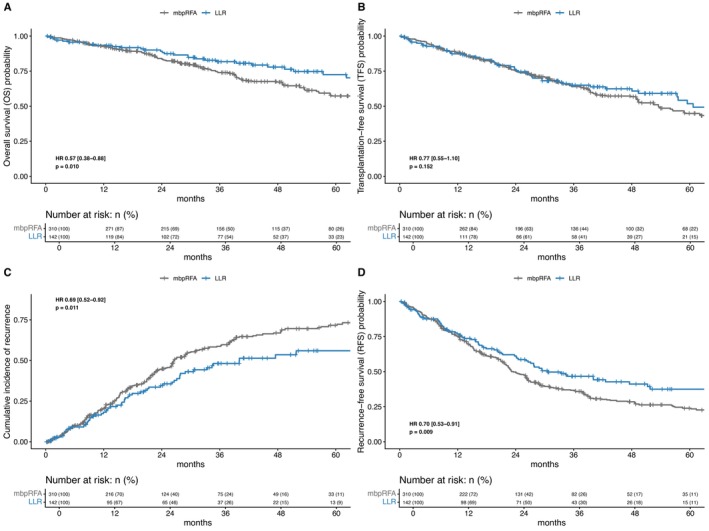
Kaplan–Meier curves in LLR versus mbpRFA patients in the matched population: Kaplan–Meier curves of (A) Overall survival, (B) Transplant‐free survival, (C) Recurrence‐free survival and (D) Cumulative incidence of recurrence in comparing LLR versus mbpRFA patients in the propensity score‐based matching. The numbers at risk were represented under the *x*‐axis. Hazard ratio (HR), confidence interval (CI 95%), and *p* value were calculated using a weighted Cox regression with matching weights. LLR, laparoscopic liver resection; mbpRFA, multibipolar radiofrequency ablation.

After matching, patients treated by LLR had better OS (HR = 0.57 [0.38, 0.88], *p* = 0.010) with no differences in TFS (HR = 0.77 [0.55, 1.1]; *p* = 0.152). Patients treated by LLR had better RFS (HR = 0.7 [0.53, 0.91]; *p* = 0.009) and TTR (HR = 0.69 [0.52, 0.92]; *p* = 0.011) (Table 3 and Figure 2). In a sensitivity analysis modelling liver transplantation as a time‐varying covariate, the protective effect of LLR on overall survival remained essentially unchanged (conditional HR 0.60, 95% CI 0.40–0.92, *p* = 0.020, versus primary HR 0.57), while liver transplantation itself was protective (HR 0.43, 95% CI 0.20–0.89, *p* = 0.024; Table S3). Among mbpRFA‐matched patients, the rate of local tumour recurrence was 14% (*n* = 43). When distant‐only recurrences were considered in the mbpRFA patients as an event, LLR was no longer significantly associated with a better RFS (*p* = 0.087) or TTR (*p* = 0.193) (Table 3).

**TABLE 3:** Long‐term outcomes according to the treatment received after propensity score matching.

Outcomes	*N* events	HR [95% CI]^a^ (mbpRFA used as reference)	*p*
**OLR vs. mbpRFA**			
Overall survival	83	1.05 [0.65,1.69]	0.853
Recurrence‐free survival	166	0.99 [0.69,1.43]	0.968
Recurrence‐free survival (distant‐only in mbpRFA patients)	160	1.13 [0.8,1.61]	0.491
Transplantation‐free survival	106	0.91 [0.56,1.47]	0.690
Time to liver transplantation	29	0.73 [0.3,1.78]	0.489
Time to recurrence	142	0.95 [0.64,1.43]	0.820
Time to recurrence (distant‐only in mbpRFA patients)	133	1.13 [0.77,1.67]	0.520
**LLR vs. mbpRFA**			
Overall survival	163	0.57 [0.38,0.88]	0.010
Recurrence‐free survival	300	0.70 [0.53,0.91]	0.009
Recurrence‐free survival (distant‐only in mbpRFA patients)	288	0.79 [0.6,1.04]	0.087
Transplantation‐free survival	213	0.77 [0.55,1.1]	0.152
Time to liver transplantation	60	1.59 [0.95,2.65]	0.077
Time to recurrence	244	0.69 [0.52,0.92]	0.011
Time to recurrence (distant‐only in mbpRFA patients)	226	0.82 [0.61,1.1]	0.193

*Note:* mbpRFA was the reference for each analysis.

Abbreviations: CI 95%, confidence interval 95%; HR, hazard ratio; LLR, laparoscopic liver resection; mbpRFA, multibipolar radiofrequency ablation; OLR, open liver resection.

^a^ Marginal HRs were estimated using a weighted (incorporating the matching weights) Cox model without covariates (i.e., non‐collapsible HR) with clustered variance on matching pair membership (cluster‐robust standard errors).

### 3.4 Propensity‐Score Matching Analysis of Patients Treated by OLR vs. mbpRFA

Using propensity‐score‐based analysis, 170 mbpRFA patients were matched to 70 OLR patients. The matched‐population descriptive analysis is shown in Table 2. In this matched population, OLR patients had increased risks of adverse events (RR = 1.69 [1.16, 2.47]; *p* = 0.007), severe adverse events (Dindo Clavien III or more, RR = 2.58 [1.16, 5.71]; *p* = 0.020), a longer hospital stay (+7 days [4–9]; *p* < 0.001) as well as an increased risk of treatment‐related mortality (RR = 4.51 [1.16, 17.59]; *p* = 0.030) compared to matched mbpRFA controls.

**FIGURE 3:**
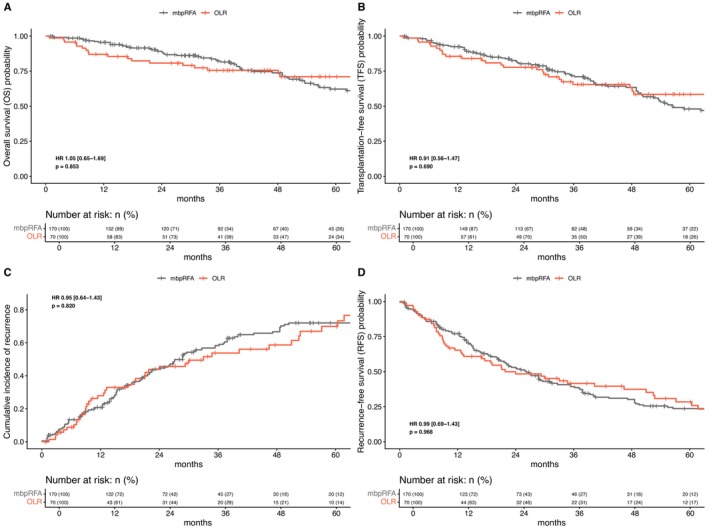
Kaplan–Meier curves in OLR versus mbpRFA patients in the matched population: Kaplan–Meier curves of (A) Overall survival, (B) Transplant‐free survival, (C) Recurrence‐free survival and (D) Cumulative incidence of recurrence in comparing OLR versus mbpRFA patients in the propensity score‐based matching. The numbers at risk were represented under the *x*‐axis. Hazard ratio (HR), confidence interval (CI 95%), and *p* value were calculated using a weighted Cox regression with matching weights. mbpRFA, multibipolar radiofrequency ablation; OLR, open liver resection.

After matching, patients treated by OLR had similar OS (HR = 1.05 [0.65, 1.69]; *p* = 0.853), TFS (HR = 0.91 [0.56, 1.47]; *p* = 0.690), or RFS (HR = 0.99 [0.69, 1.43]; *p* = 0.968) compared to mbpRFA. There was also no difference in terms of TTR (HR = 0.95 [0.64, 1.43]; *p* = 0.820) (Table 3 and Figure 3). Among mbpRFA‐matched patients, the rate of local recurrence was 11.2% (*n* = 19).

### 3.5 Sensitivity Analyses of the LLR vs. mbpRFA Main Effect on Time‐Dependent Outcomes

**FIGURE 4:**
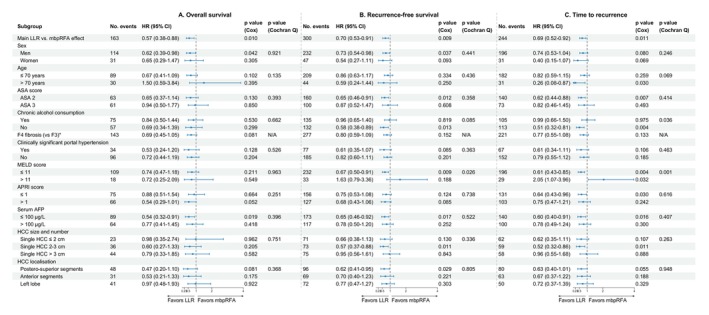
Sensitivity analyses of significant main LLR vs. mbpRFA effects within matched subpopulations. Forest plots depict the effects of LLR vs. mbpRFA (hazard ratio and 95% confidence interval) in subpopulations on (A) overall survival, (B) recurrence‐free survival, (C) time to recurrence. Hazard ratios (HR), 95% confidence intervals (CI), and *p*‐values were calculated using a weighted Cox regression with matching weights. Interaction *p*‐values are Cochran *Q* heterogeneity tests reported as exploratory; after Bonferroni correction within each endpoint, only the MELD by time‐to‐recurrence interaction remained significant (*p* = 0.012). LLR, laparoscopic liver resection; mbpRFA, multibipolar radiofrequency ablation.

Figure 4 displays forest plots of treatment effect within each pre‐specified subgroup. The LLR effect on OS was consistent across all pre‐specified subgroups (Figure 4A; all interaction Cochran Q *p* > 0.05). A similar consistency was observed for RFS, with no interaction surviving Bonferroni correction (Figure 4B). For TTR (Figure 4C), a single interaction signal was observed for MELD status (*p* = 0.012 after Bonferroni correction), with the LLR effect attenuated in patients with MELD > 11, to be interpreted as exploratory and hypothesis‐generating given the post hoc nature of subgroup analyses.

## 4 Discussion

This multicenter cohort study reported real‐world data on patients with HCC within the Milan criteria and advanced liver fibrosis (mostly cirrhosis), with diverse and common etiologies, treated exclusively with mbpRFA. It distinguishes between OLR and LLR, in contrast to many previous studies on the topic [13, 14, 15, 16, 35], and supports recent findings from the Japanese population reported in the SURF‐RCT and SURF‐cohort trials [12]. The descriptive analysis showed that the equipoise was already compromised during the study period, with mbpRFA patient profiles differing substantially from those undergoing liver resection underlining the presence of selection bias [3]. Patients who received mbpRFA were generally older and had more advanced chronic liver disease and more pronounced portal hypertension [12, 36]. Likewise, LLR seemed to be widely adopted in tertiary centres as reflected by the relatively low rate of patients undergoing OLR [18, 37]. OLR was preferred for patients with larger (> 3 cm) or technically challenging tumours (i.e., for minimally invasive surgery), reflecting the 55.1% of IMM grade 3 hepatectomies and 22% of major resections in OLR patients; consequently, OLR patients were more strictly selected, with less cirrhosis, and experienced increased post‐treatment morbidity. LLR seemed more suitable for younger patients with accessible, unique, and small tumours (≤ 3 cm). Nevertheless, LLR was still performed in a significant proportion of cirrhotic patients, including some with CSPH, as its minimally invasive nature is reported to decrease the risk of postoperative liver failure [38].

To compare treatment modalities, propensity score matching was used, including a large set of covariates to accurately reflect general status, liver functional reserve, the severity of portal hypertension, tumour burden and localization. The latter is regularly overlooked in the literature, while tumour localization contributes to technical difficulty and postoperative outcomes likely affecting treatment assignment [26]. Tumours in posterosuperior segments are known to be less accessible, especially with LLR. Both matches (LLR vs. mbpRFA or OLR vs. mbpRFA) include mostly BCLC 0/A patients, possibly transplantable, reflecting well the target population for which the question of the best first‐line treatment is still pending. LLR‐matched patients had better results than mbpRFA‐matched patients with improved OS, RFS and TTR. Still, there was no difference in TFS, and LLR patients were at increased risk of severe adverse events [35]. Surgery has been repeatedly reported to allow for better local control of HCCs, especially for tumours > 2 cm [39]. The LLR oncological benefit disappeared in this cohort when local recurrences in mbpRFA patients were overlooked. However, this distant‐only recurrence analysis was exploratory and should be interpreted cautiously. Nevertheless, the ‘local’ advantage of surgical approaches remains, therefore controversial, considering the relatively low rate of local recurrences in mbpRFA patients (< 15%) despite a substantial rate of satellite nodules (as deduced from the 29% in the LLR‐matched population), the feasibility of iterative rescue percutaneous ablations, and the fact that such patient prognosis primarily pertains to de novo HCCs [5, 12, 24, 35].

In the LLR‐matched population, most HCCs were located in the anterolateral segments, resulting in only 21.1% of grade 3 hepatectomies and 10.6% of major resections. Inversely, the OLR‐matched population included larger HCCs located in the posterosuperior segments, likely in proximity to major vasculature, which explains the increased rates of IMM grade 3 and major hepatectomies in the matched cohort (62.9% and 22.9%, respectively). This highlights the dichotomization of indications based on tumour localization and expected difficulty, with advanced LLR procedures in cirrhotic patients being more scarce and likely still limited to well‐selected profiles [21]. Consequently, OLR patients experienced significant perioperative morbidity and mortality, likely contributing to the absence of survival benefits of OLR when compared to mbpRFA patients; perioperative outcomes are indeed a key prognostic factor, also described in colorectal liver metastasis patients [18, 40]. Pre‐specified subgroup analyses (Figure 4) showed that the LLR survival benefit was consistent across all clinical and tumour‐related subgroups. The only residual signal, an attenuation of the LLR effect on TTR in patients with MELD > 11, suggests that mbpRFA may be a particularly reasonable first‐line option in patients with more advanced hepatic dysfunction, hence higher perioperative risk [41]. Despite their technical difficulty in LLR, posterosuperior localizations also seemed to challenge the efficiency of mbpRFA (decreased RFS in sensitivity analyses) as they may be associated with reduced visual control, thermal losses in proximity with sus‐hepatic veins, or increased morbidity (especially if repeated) likely contributing to their mitigated prognosis in the overall population (multivariable analyses) [7].

In France, the first‐line treatment for HCC is a comprehensive strategy that considers patient transplant eligibility and optional salvage LT [5, 42], thus transplantability and transplantation are confounders and competing events that likely influence first‐line treatment allocation and efficacy when considering OS or RFS. In such population, TFS should be considered the most relevant endpoint, especially considering the French HCC transplant strategy [43]. In this study, TFS was unaffected by the treatment type received after adjustment. Along with the favourable local results and post‐mbpRFA morbidity, this study solidifies mbpRFA as an optimized ablation technique, offering a relevant, less morbid curative alternative to surgery in cirrhotic HCC patients within Milan [23, 24] in line with the recently published SURF trial that used classical RFA method [12]. In addition, this study likely undermined the mbpRFA global efficacy as a strategy, as it did not consider the feasibility of iterative rescue percutaneous ablations [5, 36] to treat recurrences. LLR on cirrhosis remains an expert technique necessitating careful patient selection [34]; a bias, notably regarding transplant access, contributing to the positive oncological results cannot be ruled out despite matching strategies. Discrepancies in clinical profiles and potential unobserved heterogeneities (such as socio‐economic factors, alcohol withdrawal, etc.) that affect transplant eligibility limit definitive conclusions about the optimal first‐line treatment strategy within an LT allocation system. After matching, LLR patients still exhibited a trend toward a higher incidence of transplantation, which may contribute to their improved OS, while TFS does not vary. The apparent protective effect of LLR on overall survival is therefore not solely attributable to differential transplant access during follow‐up, despite the potential selection of fitter and more transplant‐eligible candidates for laparoscopic resection. This rationale also motivated the retention of TFS as a co‐primary survival endpoint alongside OS, as TFS integrates death and transplantation as a composite endpoint robust to differential transplant access between treatment arms.

Additional limitations include the study's retrospective nature and chronological bias; nevertheless, adding treatment era (2008–2012 vs. 2013–2018) as a covariate did not reveal any independent era effect on treatment allocation or on the long‐term outcomes. Furthermore, accessibility to advanced minimally invasive technologies (such as 3D laparoscopy, robotics, microwave ablation, mbpRFA, irreversible electroporation, high intensity focal ultrasound, combination of TACE and ablation), as well as local expertise and credentialing in performing these procedures, is not uniform worldwide, which may limit the generalizability of the study's findings [44, 45]. Finally, detailed causes of death and longitudinal aetiological data (such as sustained virological response after HCV therapy or HBV‐DNA suppression under nucleos(t)ide analogues) were not systematically recorded across centres, precluding the assessment of effect modification by successfully treated aetiology.

In conclusion, this study suggests that LLR is an excellent option for selected candidates. New‐generation ablative techniques such as mbpRFA offer strong alternatives with lower morbidity, maintaining optimal TFS. Minimally invasive modalities (LLR or mbpRFA) may be prioritized, when feasible, for treating/bridging HCC in Milan criteria patients with cirrhosis. Instead of focusing solely on tumour size, the treatment assignment should consider morbidity risk, tumour burden, surgical approach, technical difficulty, and localization to ensure an optimal benefit‐to‐risk ratio in attempting to maximize oncological results. As observational data, these findings do not establish the superiority of one minimally invasive modality over another and warrant confirmation in randomized trials in transplant‐eligible patients.

## Author Contributions

Contributions to conception and design: J.‐C.N., F.C., C.H., O.S. Acquisition of data: F.C., C.H., A.R., E.S., O.S., L.S., A.H., F.O., A.D.C., P.P., C.A., A.L., S.G., L.B., A.‐F.M., M.L., C.L., P.N., N.G.‐C., L.B., O.S., O.S., R.B., L.B., A.L., O.S., J.‐C.N. Analysis and interpretation of data: J.‐C.N., F.C., C.H. Drafting, revising, and the manuscript content: J.‐C.N., F.C., C.H., O.S. Final approval of the version to be published: F.C., C.H., A.R., E.S., O.S., L.S., P.P., C.A., A.L., S.G., L.B., A.‐F.M., M.L., C.L., P.N., N.G.‐C., L.B., O.S., O.S., R.B., L.B., A.L., O.S., J.‐C.N.

## Ethics Statement

This study protocol was reviewed and approved by le Comité d'éthique de la recherche en imagerie médicale (CERF‐CERIM, 47 rue de la colonie, 75 007, Paris, France), approval number [CRM‐1911‐039].

## Consent

The patients provided a written consent to participate to the study.

## Conflicts of Interest

Jean‐Charles Nault received research funding from Bayer and Ipsen. Nathalie Ganne‐Carrié received travel and congress fees, consulting fees or honoraria for lectures, presentations, speakers' bureaus from Abbvie, Gilead, and Roche. PIerre Nahon has received honoraria from and/or consults for AstraZeneca, Bayer, Bristol‐Myers Squibb, Eisai, Gilead, Guerbet, Ipsen and Roche. He received research grants from AstraZeneca, AbbVie, Bristol‐Myers Squibb and Eisai. This study was not supported by any sponsor or funder.

## Supporting information


**Table S1:** Multivariable analysis of the variables associated with the different types of treatments in the unmatched population.
**Table S2:** Multivariable analyses of the variables associated with overall survival, recurrence‐free survival, transplantation‐free survival or time to transplantation in the whole unmatched population.
**Table S3:** Sensitivity analysis of overall survival in LLR versus mbpRFA ablation, with and without modelling liver transplantation as a time‐varying covariate.
**Figure S1:** Flow chart of the study.
**Figure S2:** Graphical display of covariate balance before and after adjustment (LLR vs. mbpRFA matching).
**Figure S3:** Graphical display of covariate balance before and after adjustment (OLR vs. mbpRFA matching).

## Data Availability

The data that support the findings of this study are not publicly available (their containing information that could compromise the privacy of research participants) but are available from the corresponding author (J.‐C.N.) upon request.
