# Supplementary material for: Laparoscopic or Open Liver Resection Versus Multibipolar Radiofrequency Ablation of HCC Within Milan Criteria on Cirrhosis
Source: Liver Int. 2026 Jul 20;46(8):e70802. doi: 10.1111/liv.70802 (PMC13386131; doi:10.1111/liv.70802)
Supplement: Supplementary file 1 — Table S1: Multivariable analysis of the variables associated with the different types of treatments in the unmatched population. Table S2: Multivariable analyses of the variables associated with overall survival, recurrence‐free survival, transplantation‐free survival or time to transplantation in the whole unmatched population. Table S3: Sensitivity analysis of overall survival in LLR versus mbpRFA ablation, with and without modelling liver transplantation as a time‐varying covariate. Figure S1: Flow chart of the study. Figure S2: Graphical display of covariate balance before and after adjustment (LLR vs. mbpRFA matching). Figure S3: Graphical display of covariate balance before and after adjustment (OLR vs. mbpRFA matching). [file LIV-46-0-s001.docx]

**SUPPLEMENTARY METHODS: detailed material and methods.**

**Follow-up and right censored outcomes**

After the treatment, patients were followed up at 30 days, every three months for two years, and then every six months using either an abdominal triphasic contrast-enhanced CT scan or contrast-enhanced MRI and biology, combined with serum AFP level. In addition, chest CT was monitored every 6 months.The end of the follow-up was December 2021. The event of death was defined as death from any cause. The event recurence was defined by reccurence diagnosed at imaging and certified by multidisciplinary board staff. The transplantation of the patient listed under any pretext (i.e., liver or HCC-related indication) defined the event of liver transplantation. For mbpRFA patients, local recurrence was defined as the development of a tumor during follow-up at imaging, at the edge of the ablation zone, or within the ablation zone, encompassing treatment failure as previously defined.

The starting point for all time-dependent outcomes was the start of the first-line treatment (and first mbpRFA in case of repeated ablations). Overall Survival (OS) was measured as the time from the start of treatment until death. Recurrence-free survival (RFS) was defined as the time from the start of treatment to either disease recurrence or death. Liver transplantation during follow-up was considered a non-event for these two outcomes, and patients were followed up after liver transplantation until death or the date of last follow-up for the patients still alive.

Time to Liver Transplantation (TLT) was calculated as the duration from the start of treatment to the date of liver transplantation. Transplantation-free survival (TFS) is the time from the start of treatment to either liver transplantation or death, whichever occurs first, recurrence being considered as non-event.

Time to recurrence (TTR) was defined as the duration until disease recurrence, with patients who underwent liver transplantation (LT) or died before recurrence being censored. For this endpoint, both liver transplantation and death were treated as competing risks, and the computed hazard ratios (HR) were cause-specific HRs.

Percutaneous ablation has been reported to offer the possibility of optimizing local control of HCCs through iterative ablation of local recurrences when they occur. Therefore, the investigators of this study also decided to explore a scenario with an alternative endpoint for recurrence, excluding local recurrences and considering distant-only ones as an event in the mbpRFA arm.

All computed HRs represent the ratio of hazard rates between two groups, reflecting the instantaneous risk of an event occurring. An HR < 1 indicates a lower risk of the event, suggesting better survival or longer time to the event, while an HR > 1 indicates a higher risk and poorer survival.

**Statistical analysis**

In this study, the exposure variable was the first-line treatment modality (LLR or OLR vs. mbpRFA). The primary objective was to define the effect of the treatment modality on long-term outcomes such as OS and RFS.

Continuous data were expressed as median (25-75 inter-quartiles), were not categorized, and were compared using the Mann-Whitney U test or Kruskal-Wallis test, as appropriate. Categorical data are expressed as percentages and were compared using Pearson's chi-square test or Fisher's exact test, as appropriate. Statistical significance testing was 2-sided. Survival probabilities were computed using the Kaplan-Meier estimate and compared using the log-rank test. A p-value <0.05 was considered statistically significant for all tests. Missing values are reported in **Table 1,** and no variables had >12% of missing values. All models were checked for singularity, convergence, autocorrelation, and collinearity where appropriate.

Age was transformed in the regressions using natural splines with three degrees of freedom to avoid non-linear relationship misspecification due to the non-normal distribution. Serum Alphafoetoprotein (UI/L) was transformed in regressions using its common logarithm. The size and number of HCCs were combined into a 4-level categorical variable for clinical relevance as follows: very early HCCs (single HCCs ≤2 cm), single HCCs 2-3 cm, single HCCs >3 cm, and multiple HCCs (maximum 3). HCC localization was a 3-level categorical variable coded as posterosuperior, anterior, or left lobe localisations.

A set of clinically relevant variables was considered to require balance (i.e., adjustment variables) as they likely are associated with the treatment arm or outcomes of interest. The variables included in the propensity score were HCC size/number, gender, age, ASA, HCV, HBV, metabolic syndrome, chronic alcohol intake, MELD score, serum AFP level, APRI score, cirrhosis, CSPH, and HCC localization. These variables were included systematically in all multivariable models and served as covariates in matching analyses.

Right-censored outcomes regressions were analyzed using Cox proportional-hazards regression models (estimated effect sizes were conditional Hazard Ratio (HR) with a 95% confidence interval (CI 95%)). Binary outcomes regressions were logistic regressions (estimated effect sizes were Odd’s Ratio (OR) with CI 95%). Both types of regressions were performed using cluster-center robust variance to account for inter-center variability.

The models of exposure were propensity score-based matched analyses. All matching was performed using a 3:1 nearest neighbor matching without replacement using propensity score distance (estimated with probit regression) and a caliper <0.1 to estimate marginal effects (i.e., average effect of exposure on the population). The target estimation was the average treatment effect on the treated. The balance of the matching variables before and after matching was estimated using standardized mean differences (**Supplementary figures 2 and 3**). Sensitivity analyses were conducted by applying the same propensity score matching procedure (excluding the covariate used for subgroup definition) within subpopulations defined by the covariates included in the main propensity score model.

Marginal effects were computed using the g-computation method. For continuous or binary endpoints, marginal differences or log risk ratio with 95%CI were computed using a weighted linear or logistic regression incorporating matching weights and clustered variance on matching pair membership to account for cluster-robust standard errors. These regressions included the outcome of interest as the response variable and, as independent variables, the treatment arm, the quantile distribution of the distance, and their interaction. Relative risks were obtained for binary variables by exponentiating the marginal log risk ratios. Marginal HRs were estimated using a weighted (incorporating the matching weights) Cox model without covariates (i.e., non-collapsible HR) with clustered variance on matching pair membership (cluster-robust standard errors). All statistical analyses were performed by C.H. using R statistical software version R version 4.2.0.

| **Supplementary Table S1: Multivariable analysis of the variables associated with the different types of treatments in the unmatched population.** | | | | | | |
| --- | --- | --- | --- | --- | --- | --- |
| **Variable** | **Multibipolar radiofrequency ablation vs. other treatments** | | **Laparoscopic liver resection vs. other treatments** | | **Open liver resection vs. other treatments** | |
|  | **Odds ratio [95% confidence interval]** | **P value** | **Odds ratio [95% confidence interval]** | **P value** | **Odds ratio [95% confidence interval]** | **P value** |
| Gender (Male) | 0.89[0.56,1.41] | 0.605 | 1.20[0.71,2.04] | 0.500 | 1.09[0.65,1.82] | 0.737 |
| Age (years) > 25th percentile † | 0.88[0.69,1.11] | 0.279 | 1.27[0.81,2.00] | 0.300 | 0.90[0.50,1.62] | 0.731 |
| Age (years) 25 to 75th percentile † | 0.98[0.78,1.24] | 0.873 | 0.86[0.67,1.10] | 0.226 | 1.42[0.96,2.10] | 0.083 |
| Age (years) > 75th percentile † | 2.69[1.66,4.36] | <0.001 | 0.50[0.38,0.66] | <0.001 | 0.41[0.15,1.13] | 0.085 |
| ASA grade 3 (vs. 2) | 1.03[0.14,7.52] | 0.978 | 0.97[0.19,4.92] | 0.973 | 0.87[0.23,3.23] | 0.830 |
| Chronic HCV | 0.65[0.40,1.05] | 0.077 | 1.66[0.89,3.10] | 0.111 | 1.12[0.42,2.97] | 0.819 |
| Chronic HBV | 0.40[0.16,0.95] | 0.038 | 2.45[0.95,6.30] | 0.063 | 1.52[0.46,5.01] | 0.490 |
| Metabolic syndrome | 1.50[0.68,3.31] | 0.317 | 0.83[0.43,1.59] | 0.566 | 0.75[0.24,2.32] | 0.618 |
| Chronic alcohol intake | 1.07[0.50,2.30] | 0.864 | 1.12[0.51,2.43] | 0.776 | 0.76[0.33,1.79] | 0.537 |
| MELD score | 1.09[0.92,1.29] | 0.331 | 1.00[0.87,1.16] | 0.972 | 0.81[0.70,0.92] | 0.002 |
| APRI score | 0.99[0.93,1.06] | 0.834 | 0.97[0.91,1.02] | 0.220 | 1.06[0.93,1.22] | 0.376 |
| Single HCC of ≤2cm | *Reference* | — | *Reference* | — | *Reference* | — |
| 2cm < Single HCC ≤ 3cm | 0.94[0.62,1.42] | 0.756 | 0.86[0.61,1.22] | 0.397 | 1.68[1.12,2.52] | 0.012 |
| Single HCC > 3cm | 0.41[0.20,0.82] | 0.012 | 1.71[0.85,3.43] | 0.135 | 2.44[1.44,4.15] | <0.001 |
| 2 or 3 HCCs, ≤ 3cm | 1.49[1.00,2.21] | 0.052 | 0.53[0.39,0.74] | <0.001 | 1.30[0.69,2.47] | 0.413 |
| Serum AFP (log 10) | 0.84[0.71,0.99] | 0.040 | 1.09[0.97,1.22] | 0.146 | 1.14[0.88,1.47] | 0.312 |
| F4 fibrosis (vs. F3) | 6.84[3.02,15.51] | <0.001 | 0.34[0.14,0.82] | 0.017 | 0.36[0.18,0.70] | 0.003 |
| HCC localisation: Postero superior | *Reference* | — | *Reference* | — | *Reference* | — |
| HCC localisation: Anterior | 1.29[0.88,1.89] | 0.193 | 1.24[0.67,2.28] | 0.500 | 0.43[0.26,0.70] | <0.001 |
| HCC localisation: Left lobe | 0.56[0.34,0.93] | 0.024 | 3.37[2.31,4.93] | <0.001 | 0.28[0.13,0.61] | 0.001 |
| CSPH | 2.94[1.55,5.60] | 0.001 | 0.51[0.25,1.03] | 0.062 | 0.23[0.13,0.39] | <0.001 |
| Treatment era (2013-2018 vs. 2008-2012) | 0.80[0.16,4.00] | 0.783 | 1.91[0.37,9.81] | 0.440 | 0.52[0.21,1.31] | 0.167 |
| *Logistic regressions with center-specific robust variance to account for inter-center variability, including the treatment arm (one versus the rest) as the response variable. All models included 849 observations. † Non-linear age was modelled using cubic spline functions with three degrees of freedom; knots were placed at the 25th and 75th percentiles of the variable in the overall population. Treatment era was added as an additional binary covariate (2013-2018 versus 2008-2012, with patients enrolled before 2008 grouped with the earlier era and patients enrolled in 2019 grouped with the later era).* | | | | | | |

| **Supplementary Table S2: Multivariable analyses of the variables associated with overall survival, recurrence-free survival, transplantation-free survival or time to transplantation in the whole unmatched population** | | | | | | | | |
| --- | --- | --- | --- | --- | --- | --- | --- | --- |
| **Variable** | **Overall survival** | | **Recurrence-free survival** | | **Transplantation-free survival** | | **Time to liver transplantation** | |
|  | **Hazard ratio [95% CI]** | **P value** | **Hazard ratio [95% CI]** | **P value** | **Hazard ratio [95% CI]** | **P value** | **Hazard ratio [95% CI]** | **P value** |
| **Treatment** |  |  |  |  |  |  |  |  |
| Multibipolar radiofrequency ablation | *Reference* | — | *Reference* | — | *Reference* | — | *Reference* | — |
| Laparoscopic liver resection | 0.75[0.54,1.03] | 0.078 | 0.79[0.65,0.96] | 0.017 | 0.90[0.73,1.11] | 0.327 | 1.24[0.73,2.10] | 0.428 |
| Open liver resection | 1.00[0.67,1.49] | 0.999 | 1.08[0.84,1.39] | 0.559 | 0.92[0.71,1.19] | 0.533 | 0.90[0.52,1.56] | 0.695 |
| **HCC size and number** |  |  |  |  |  |  |  |  |
| Single HCC of ≤2cm (BCLC 0) | *Reference* | — | *Reference* | — | *Reference* | — | *Reference* | — |
| 2cm < Single HCC ≤ 3cm | 1.11[0.88,1.39] | 0.387 | 1.17[0.96,1.43] | 0.117 | 0.99[0.79,1.23] | 0.901 | 0.76[0.41,1.39] | 0.370 |
| Single HCC > 3cm | 1.45[1.17,1.81] | <0.001 | 1.42[1.28,1.58] | <0.001 | 1.61[1.21,2.16] | 0.001 | 1.30[0.82,2.07] | 0.270 |
| 2 or 3 HCCs, ≤ 3cm | 1.17[0.87,1.56] | 0.297 | 1.22[1.00,1.50] | 0.051 | 1.53[1.23,1.92] | <0.001 | 2.42[1.74,3.37] | <0.001 |
| Gender (Male) | 0.79[0.58,1.07] | 0.126 | 0.94[0.75,1.19] | 0.629 | 0.88[0.73,1.04] | 0.137 | 1.82[1.19,2.78] | 0.006 |
| Age (years) > 25th percentile † | 2.57[0.27,24.45] | 0.411 | 1.25[0.51,3.10] | 0.625 | 0.51[0.18,1.44] | 0.207 | 0.98[0.27,3.55] | 0.971 |
| Age (years) 25 to 75th percentile † | 5.37[0.01,2484] | 0.591 | 2.60[0.07,91.6] | 0.598 | 0.54[0.02,12.44] | 0.703 | 0.00[0.00,0.01] | 0.014 |
| Age (years) > 75th percentile † | 7.99[2.02,31.54] | 0.003 | 1.69[0.52,5.44] | 0.380 | 1.49[0.53,4.22] | 0.451 | 0.00[0.00,0.00] | 0.001 |
| ASA grade 3 (vs. 2) | 1.23[0.84,1.81] | 0.283 | 1.08[0.96,1.21] | 0.181 | 1.09[0.81,1.47] | 0.583 | 0.66[0.54,0.80] | <0.001 |
| Chronic HCV | 0.97[0.84,1.13] | 0.719 | 1.01[0.78,1.32] | 0.917 | 0.81[0.68,0.97] | 0.020 | 0.54[0.39,0.73] | <0.001 |
| Chronic HBV | 0.85[0.59,1.23] | 0.390 | 0.70[0.51,0.95] | 0.022 | 0.80[0.60,1.08] | 0.140 | 0.55[0.32,0.93] | 0.025 |
| Metabolic syndrome | 1.13[0.85,1.49] | 0.398 | 1.15[0.92,1.44] | 0.223 | 1.00[0.70,1.43] | 0.982 | 0.75[0.51,1.10] | 0.137 |
| Chronic alcohol intake | 1.38[1.13,1.69] | 0.002 | 1.06[0.85,1.33] | 0.590 | 0.97[0.83,1.15] | 0.757 | 0.49[0.40,0.60] | <0.001 |
| MELD score | 1.12[1.09,1.15] | <0.001 | 1.07[1.05,1.09] | <0.001 | 1.13[1.09,1.16] | <0.001 | 1.11[1.06,1.17] | <0.001 |
| APRI score | 1.04[1.02,1.06] | <0.001 | 1.00[0.98,1.02] | 0.846 | 1.03[1.02,1.04] | <0.001 | 1.07[1.04,1.10] | <0.001 |
| Serum AFP (log 10) | 1.29[1.14,1.46] | <0.001 | 1.27[1.09,1.46] | 0.002 | 1.20[1.11,1.30] | <0.001 | 1.07[0.88,1.29] | 0.494 |
| CSPH | 1.11[0.98,1.26] | 0.114 | 1.19[1.05,1.36] | 0.008 | 1.34[1.18,1.51] | <0.001 | 1.54[1.20,1.97] | <0.001 |
| F4 fibrosis (vs. F3) | 3.19[2.05,4.96] | <0.001 | 1.63[1.07,2.47] | 0.023 | 1.66[0.80,3.42] | 0.174 | 0.61[0.36,1.04] | 0.068 |
| **HCC localisation** |  |  |  |  |  |  |  |  |
| Postero superior | *Reference* | — | *Reference* | — | *Reference* | — | *Reference* | — |
| Anterior | 0.84[0.72,0.97] | 0.021 | 1.10[0.96,1.26] | 0.170 | 0.85[0.73,0.98] | 0.027 | 1.00[0.72,1.38] | 0.992 |
| Left lobe | 1.13[0.91,1.40] | 0.275 | 1.12[0.97,1.28] | 0.126 | 1.10[0.84,1.42] | 0.492 | 1.29[0.83,2.00] | 0.262 |
| Treatment era (2013-2018 vs. 2008-2012) | 0.81[0.61,1.08] | 0.150 | 0.99[0.86,1.16] | 0.942 | 0.96[0.85,1.08] | 0.512 | 1.26[0.87,1.83] | 0.218 |
| *Cox proportional hazards regressions with center-specific robust variance to account for inter-center variability. All models included 849 observations (overall survival 286 events, recurrence-free survival 549, transplantation-free survival 377, time to liver transplantation 110). † Non-linear age was modelled using cubic spline functions with three degrees of freedom; knots were placed at the 25th and 75th percentiles of the variable in the overall population. Treatment era was added as an additional binary covariate (2013-2018 versus 2008-2012, with patients enrolled before 2008 grouped with the earlier era and patients enrolled in 2019 grouped with the later era).* | | | | | | | | |

| **Supplementary Table S3: Sensitivity analysis of overall survival in LLR versus mbpRFA ablation, with and without modelling liver transplantation as a time-varying covariate** | | | |
| --- | --- | --- | --- |
|  | **Hazard ratio for LLR vs. mbpRFA** | **95% Confidence interval** | **P value** |
| Primary Cox model (matched cohort, cluster on matching pairs, marginal HR) | 0.57 | 0.38-0.88 | 0.010 |
| Cox model with liver transplantation as time-varying covariate (conditional HR) | 0.60 | 0.40-0.92 | 0.020 |
| Cox proportional hazards models on the matched LLR versus mbpRFA cohort (n=452, 142 matching pairs, 163 deaths). Models incorporated matching weights, with standard errors clustered on matching pairs (marginal hazard ratios, consistent with Table 3). The time-varying covariate model used liver transplantation as a time-dependent indicator switching from 0 to 1 at the date of transplantation. Sixty patients (13.3%) underwent liver transplantation during follow-up. In this model, liver transplantation itself was associated with reduced mortality (hazard ratio 0.43, 95% CI 0.20-0.89, p=0.024), as expected, while the LLR effect remained essentially unchanged (conditional HR 0.60 versus primary 0.57). The imbalance in cumulative transplantation incidence between arms was not statistically significant within the matched cohort (p=0.077). | | | |

**SUPPLEMENTARY FIGURES**

**Supplementary Figure 1:** **Flow chart of the study**

**
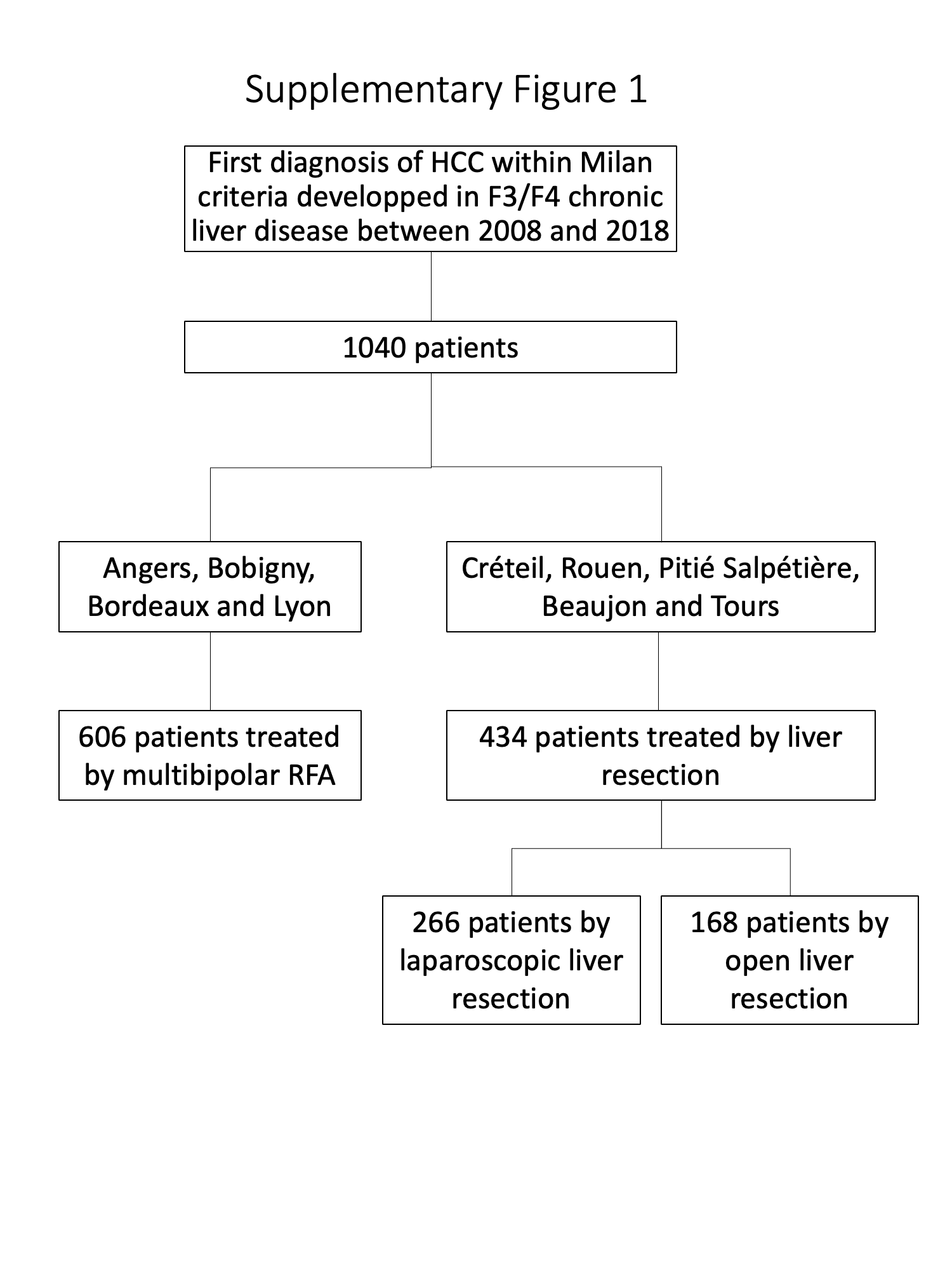
**

**Supplementary Figure 2: Graphical display of covariate balance before and after adjustment (LLR vs mbpRFA matching).**

**
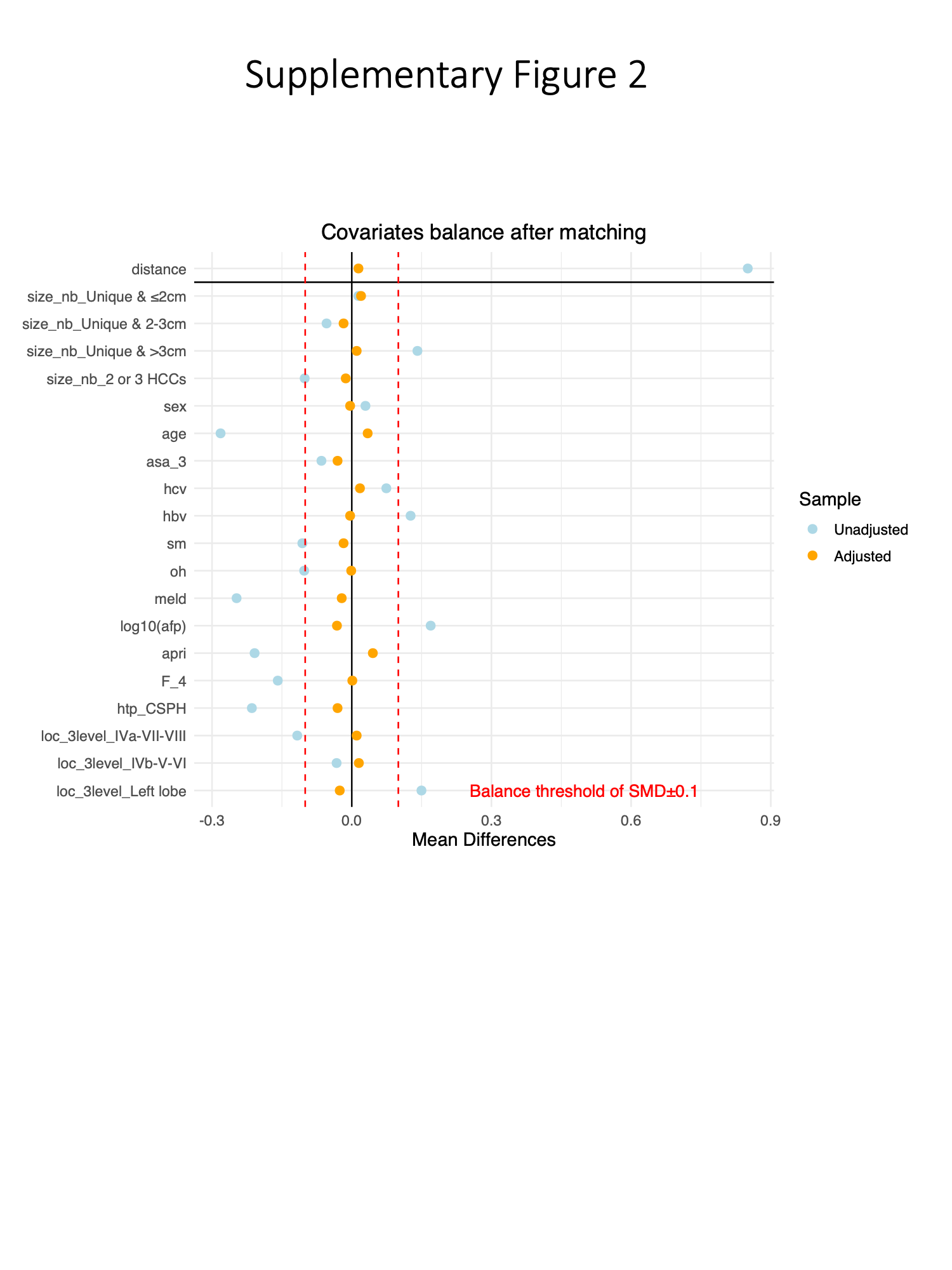
**

**Supplementary Figure 3:** **Graphical display of covariate balance before and after adjustment (OLR vs mbpRFA matching).**

**
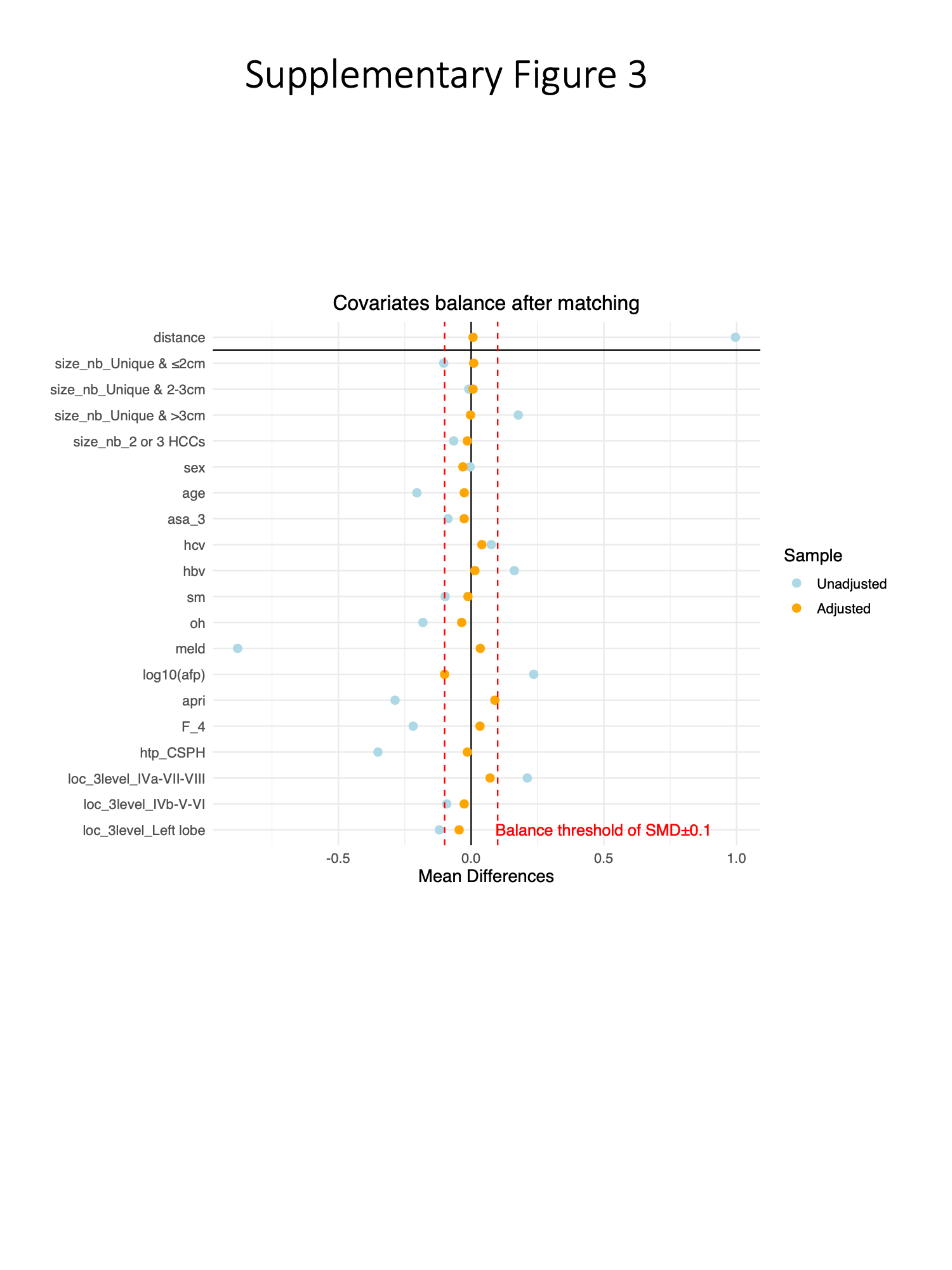
**
